# 

**Published:** 1895-04

**Authors:** 


### CONTENTS.

ORIGINAL ARTICLES—

A paper read before the Odontological Society, of Atlanta, Ga., at the December meeting. By Dr. D. S. Arnold, Atlanta, Ga. 181

Address of J. McF. Gaston, M. D., before the Graduates of the Southern Medical College, April 3d, 1895 ..... 186

The Treatment of Empyema of the Antrum of Highmore. By Robert Levy, M. D., Denver, Col. ..... 195

Report of a Case of Congenital Cyanosis, With Remarks. By H. W. McLauthlin, M. D., Denver, Col ..... 199

The Local Treatment of Acute Follicular Tonsillitis. By A. B. Farnham, M. D., of Milwaukee ..... 206

Complicated Herniæ. By Dr. S. E. Milliken, of New York ..... 207

SELECTIONS AND ABSTRACTS—

Trional ..... 208

Quinine in Chorea ..... 208

Bleaching teeth. D. D. Wiber, D. D. S., Washington ..... 209

Rupture of the Uterus During Labor. L. M. Bossi, Geneva ..... 210

The real value of the Medicinal Peroxide of Hydrogen Preparations found in the market. By H. Endemann, Ph. D., chemist 213

Medical Impertinence ..... 214

The Use of the Murphy Button. J. B. Murphy, Chicago ..... 215

Fistula in Ano. Joseph B. Bacon, Chicago ..... 216

A Unique Malformation of the Urethra, associated with Piles. Napoleon J. Bloomfield, Ohio ..... 219

SOCIETY REPORTS—

Richmond Academy of Medicine and Surgery. March, 1895. Mark W. Peyser, M. D., Sec'y .......... 220

BOOK REVIEWS—

Home Ereatment for Catarrhs and Colds—Leonard A. Dessar, M. D .......... 225
An Outline of the Embryology of the Eye—Prize Essay. By Mark A. Halden, A. M., M. D .......... 225
A Manual of Bandaging—Adapted for Self Instruction. By C. Henri Leonard, A. M., M. D .......... 225
Syllabus of Gynæcology Based on The American Text Book of Gynæcology. By J. W. Long, M. D., Richmond .......... 226
Notes on the Newer Remedies, Their Therapeutic Application and Modes of Administration. By David Cerna, M. D., Ph. D. .......... 226
Laboratory Guide for the Bacteriologist. Langdon Frothingham, M. D. V .......... 227
A Book of Detachable Diet Lists; for Albuminuria, etc., etc. Jerome B. Thomas, A. B., M. D .......... 227
Dose Book and Manual of Prescription Writing. E. D. Thornton, M. D., Ph. G .......... 227

EDITORIAL—

Atlanta Medical College .......... 228
Business Department .......... 231
Prescription Department .......... 232
Special Notes .......... 230

